# The significance of glial cell line-derived neurotrophic factor analysis in Progressive Supranuclear Palsy

**DOI:** 10.1038/s41598-024-53355-y

**Published:** 2024-02-02

**Authors:** Piotr Alster, Dagmara Otto-Ślusarczyk, Stanisław Szlufik, Karolina Duszyńska-Wąs, Agnieszka Drzewińska, Alicja Wiercińska-Drapało, Marta Struga, Michał Kutyłowski, Andrzej Friedman, Natalia Madetko-Alster

**Affiliations:** 1 Department of Neurology, Medical University of Warsaw, Kondratowicza 8, 03-242 Warsaw, Poland; 2 Department of Biochemistry, Medical University of Warsaw, Warsaw, Poland; 3 Department of Hepatology and Infectious and Tropical Diseases, Medical University of Warsaw, Provincial Infectious Diseases Hospital in Warsaw, Warsaw, Poland; 4 Department of Diagnostic Imaging, Brodno Mazovian Hospital, Warsaw, Poland

**Keywords:** Parkinson's disease, Parkinson's disease

## Abstract

Progressive Supranuclear Palsy (PSP) is an atypical parkinsonism. Major subtypes of the disease: PSP-Richardson’s Syndrome (PSP-RS) and PSP Parkinsonism Predominant (PSP-P) vary in clinical features, the pathomechanism remains unexplored. The aim of this work is to analyze the relevance of glial cell line-derived neurotrophic factor (GDNF) evaluation in the serum and cerebrospinal fluid (CSF) in PSP subtypes and to verify its significance as a possible factor in the in vivo examination. Authors assessed the concentration of GDNF in the serum and CSF of 12 patients with PSP-RS, 12 with PSP-P and 12 controls. Additionally authors evaluated patients using Unified Parkinson’s Disease Rating Scale—III part (UPDRS-III), Frontal Assessment Battery (FAB) and Magnetic Resonance Imaging (MRI). The evaluation revealed significantly increased concentrations of GDNF in the CSF among PSP-RS patients and substantially increased concentrations of GDNF in the serum in PSP-P. Though the GDNF concentrations differentiated PSP subtypes, no correlations between with clinical factors were observed however certain correlations with atrophic changes in MRI were detected. GDNF is a factor which may impact the pathogenesis of PSP. Possible implementation of GDNF as a therapeutic factor could be a perspective in the search for therapy in this currently incurable disease.

## Introduction

Progressive Supranuclear Palsy is a tauopathic atypical parkinsonism defined by the Höglinger et al. diagnostic criteria^1^. It is a clinical entity characterized by such features as akinesia, oculomotor dysfunction, cognitive and speech deterioration and postural instability^1^. The most common phenotypes of this entity are Progressive Supranuclear Palsy—Richardson Syndromes (PSP-RS) and Progressive Supranuclear Palsy—Parkinsonism Predominant (PSP-P). The two major phenotypes are associated with up to 90% of cases of PSP^2^. Less common phenotypes such as PSP-Frontal (PSP-F), PSP with initial predominance of oculomotor dysfunction (PSP-OM), PSP with initial predominance of primary lateral sclerosis (PSP-PLS), PSP with initial predominance of speech/language disorders (PSP-SL), PSP with initial predominance of cerebellar ataxia (PSP-C), PSP with initial predominance of corticobasal syndrome (PSP-CBS), non-fluent/agrammatic primary progressive aphasia(nfaPPA) and PSP with initial predominance of postural instability (PSP-PI) are briefly described in literature. The major two phenotypes relevantly differ in the context of the clinical course and disease duration^1^. The differentiation using biomarkers has not been explored. However certain factors such as neurofilament light chain in the cerebrospinal fluid (CSF) and plasma were found to be associated with the progression of the disease in PSP^3^. Growing interest is linked with the distribution of tau and microglial activation in both PSP phenotypes^4^. The dissemination of tau and neuronal loss was interpreted as possibly impacting the pathological and clinical diversity in PSP. The analyses of radiotracers in positron emission tomography (PET) is affected by limited access to it and high cost. The evaluation of inflammatory parameters and their possibly differential impact is an evolving issue. However, less is known in the context of neurotrophic factors such as glial cell line-derived neurotrophic factor (GDNF). The compound was previously examined in more common neurodegenerative diseases such as Alzheimer’s Disease and Parkinson’s Disease (PD)^5^. The aim of the study is to determine if GDNF concentrations in the serum and CSF differ between PSP-RS and PSP-P phenotypes. Additionally authors intended to verify the significance of the role of this factor.

## Material and methods

### Patient recruitment

The study is based on the evaluation of 12 patients with PSP-RS (7 males and 5 females) aged 64–75 (mean age 70 ± 3.6) 12 patients with PSP-P (7 males and 5 females) aged 55–80 (mean age 68.8 ± 6.7), and 12 healthy volunteers (7 males and 5 females) aged 35–69. The diagnosis of PSP was based on the recent criteria of diagnosis. The examination was done by neurologists experienced in movement disorders^1^.

### Inclusion and exclusion criteria

The duration of the disease varied from 3 to 6 years. The mean duration of the disease in PSP-P was 3.5 years, whereas in PSP-P 4.5 years. Among the exclusion criteria authors indicated cancer, infectious diseases, stroke in the past and autoimmunological diseases. None of the patients was diagnosed with diabetes. None of the patients used drugs, which according to characteristics of medicinal product or literature were found to be impacting the level of GDNF. All patients with PSP were examined in the Department of Neurology of the Medical University of Warsaw, whereas healthy volunteers were assessed in the Department of Infectious Diseases, Tropical Diseases and Hepatology of the Medical University of Warsaw. All patients affected by PSP were examined in “OFF” stage. Additionally, to extend the clinical overview of the assessment each patient was evaluated using the third part of the Unified Parkinson’s Disease Rating Scale (UPDRS-III). The UPDRS-III is a validated method of assessment enabling evaluation of motor impairments^6^. Moreover, based on previous studies performed by the research group, each patient was examined by a neuropsychologist using the Frontal Assessment Battery (FAB)^7^. The FAB has been developed as a brief test of executive function that can be administered at the bedside. The method consists of six subtests. Each of them is able to examine a specific cognitive or behavioral domain related to the frontal lobes such as: verbal conceptualization, verbal fluency, motor programming, sensitivity to interference, inhibitory control and environmental autonomy. Low score indicates executive dysfunction. The FAB is broadly used as a tool for assessment of executive function and may provide useful information for differential diagnosis in several diseases^8–10^. The healthy volunteers were negatively verified in the context of infection, diabetes mellitus and neurological deficits. The healthy volunteers did not use any drugs impacting the level of GDNF.

### CSF and blood collection procedures

All of the patients included in the study underwent lumbar puncture and blood samples were taken. From each patient 10 ml of CSF and 10 ml of serum were analyzed. The samples of serum were placed in tubes without anticoagulant. The serum and CSF obtained in the study were frozen at − 80 °C until evaluation.

### MRI evaluation

All of the patients in the study underwent a magnetic resonance imaging (MRI) using the Siemens 3.0T device and the examinations were evaluated by a radiologist with an experience of more than 5 years in neuroimaging using a dedicated software. All the measurements were obtained in T2-weighted sequences, the area of the pons (P) and the midbrain (M) in the midsagittal plane, the average width of middle cerebellar peduncles (MCP) in the sagittal plane, the average width of superior cerebellar peduncles (SCP) in the coronal plane, the average width of the third ventricle (V3) and maximal width of the frontal horns of the lateral ventricles (FH) in the axial plane. The magnetic resonance parkinsonism index (MRPI) was calculated based on the formula MRPI = (P/M) × (MCP/SCP), whereas the magnetic resonance parkinsonism index 2.0 (MRPI 2.0) on the formula MRPI 2.0 = MRPI × (V3/FH)^11,12^.

### Biomarker estimation

The concentrations of GDNF and tau were assessed in the material. Tau concentration was analyzed in the cerebrospinal fluid only. GNDF and tau were measured using commercial ELISAs kits (GDNF ELISA kits from Diaclon SAS, and the Tau protein ELISA kit from Cloud-Clone Corp). Absorbance was determined at 450 nm using a plate reader. The concentrations of the tested markers were calculated based on the standard curve.

### Statistical analysis

The results revealed in the analysis were statistically evaluated using the GraphPad Prisma 8 program. Arithmetic means (X) with standard deviations (SD) were assessed. In assessing the statistical significance of differences between the means, the authors indicated a degree of significance of P < 0.05, applicable statistical tests were used. To obtain the distribution (evaluation of normality) of the analyzed variables, the Shapiro–Wilk W test was utilized in the work. Based on the distribution revealed in this evaluation, the parametric t-test or the non-parametric Mann–Whitney test were used to obtain a comparison of the distributions of the variable in the two groups. An ANOVA test was used to compare the mean in many groups.

### Ethical approval

The studies involving humans were approved by the Bioethical Committee of Medical University of Warsaw—approval numbers: KB/139/2020 and KB/1243/2016. The studies were conducted in accordance with the local legislation and institutional requirements. The participants provided their written informed consent to participate in this study. The study was performed in accordance with the Declaration of Helsinki.

## Results:

The total of 24 PSP patients were included and classified into two groups: PSP-RS with Richardson syndrome (n = 12), patients with Parkinsonism Predominant (PSP-P, n = 12) and 12 healthy controls whose clinical data as well as FAB and UPDRS III are shown in Tables 1 and 2. There were no significant differences in the age and sex ratio level among two PSP groups (*P* > 0.05). Authors also compared the clinical symptoms (FAB and UPDRS -III grades) and there was a significant difference between the PSP- RS patients and PSP-P (*P* < 0.02 FAB; *P* < 0.01—UPDRS-III). In the present study, the GDNF concentrations in CSF and serum were investigated among patients with Progressive Supranuclear Palsy (PSP).

**Table 1:** Clinical data of the study group.

Variables	Patients with PSP-RS (n = 12)	Patients with PSP-P (n = 12)	Controls (n = 12)	P-value*
	Mean ± SD	Mean ± SD	Mean ± SD	
Sex (female/male)	5/7	5/7	7/5	–
Age [y]	70 ± 3.6	68.8 ± 6.7	50 ± 8.8	0.0001^a^
Average disease duration [years]	3.5	4.5	–	
FAB	9.6 ± 2.6	13.5 ± 3.6	–	0.02^b^
UPDRS III	41 ± 9.4	27.2 ± 10.4	–	0.01^b^

PSP-(RS), Progressive Supranuclear Palsy—Richardson Syndrome; PSP-(P), Progressive Supranuclear Palsy-Parkinsonism Predominant; FAB, frontal assessment battery; UPDRS-III, Unified PD rating scale part III exercise evaluation.

*Significant *P*-value (*α* = 0.05).

^a^Significant for PSP (RS) or (P) × control.

^b^Significant for PSP-RS × PSP-P.

**Table 2:** Basic information concerning the groups examined in the study.

	PSP-P (n = 12)	PSP-RS (n = 12)	Healthy controls (n = 12)
Level of education of the patients	Higher—4/12	Higher—1/12	Higher—3/12
	Secondary—8/12	Secondary—10/12	Secondary—9/12
		Primary—1/12	
Non-motor symptoms observed among patients in the group	Increased salivation	Constipation	None
	Drooling	Dysosmia	
	Constipation	Dyssomnia	
	Seborrhea	Voiding disorders	
	Voiding disorders		
Comorbidities observed among patients	Hypertension	Hypertension	Hypertension
	Heart arythmia	Heart arythmia	Back pain
	Obstructive sleep	Inguinal hernia	
	Apnea	Back pain	
	Glaucoma		
Neurodegenerative diseases in the families of the patients	1 patient—mother with MSA	None	None
	Other patients—None		

PSP-RS, Progressive Supranuclear Palsy—Richardson Syndrome; PSP-P, Progressive Supranuclear Palsy-Parkinsonism Predominant; MSA, multiple system atrophy.

**Figure 1:**
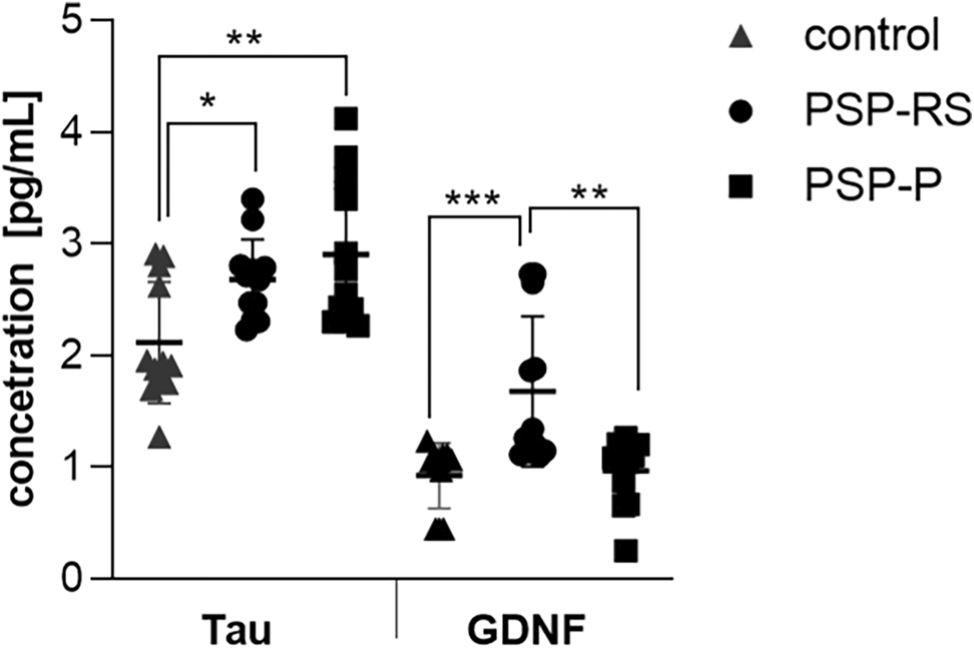
The analysis of Tau and GDNF in CSF. Levels of Tau and GDNF were measured by ELISA test in CSF from PSP-RS and PSP-P in comparison to control. Data are expressed as the mean ± SD performed in duplicates. Statistical significance was calculated using the Mann–Whitney U-test. * P < 0.01, ** P < 0.001; *** P < 0.0001.

**Figure 2:**
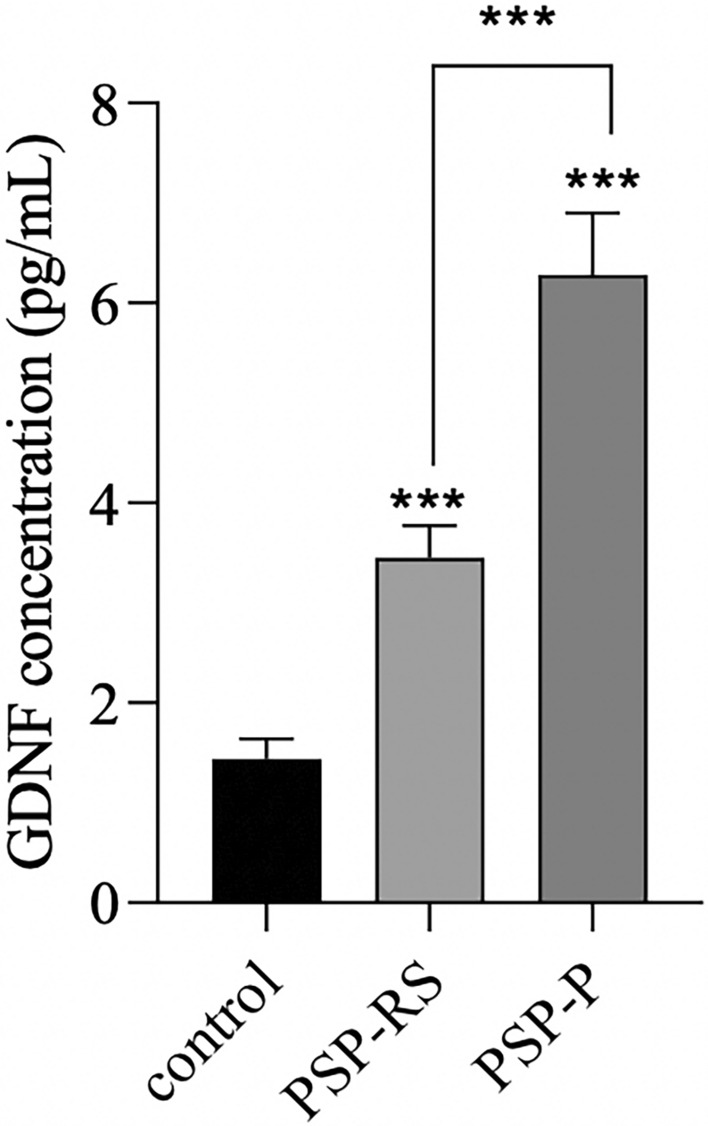
The analysis of GDNF in serum. Levels of GDNF were measured by ELISA test in serum from PSP-RS and PSP-P in comparison to control. Data are expressed as the mean ± SD performed in duplicates. Statistical significance was calculated using the Mann–Whitney *U*-test. *** *P* < 0.0001.

**Figure 3:**
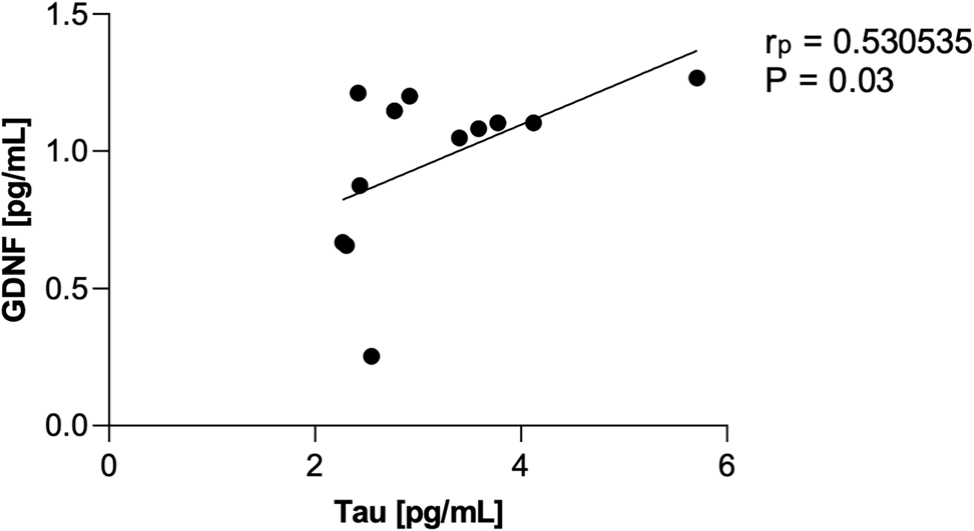
Correlation analysis cerebrospinal fluid (CSF) levels of Tau vs GDNF in Progressive Supranuclear Palsy with predominant parkinsonism (PSP) patients; determined using Pearson’s correlation coefficient (rp).

**Figure 4:**
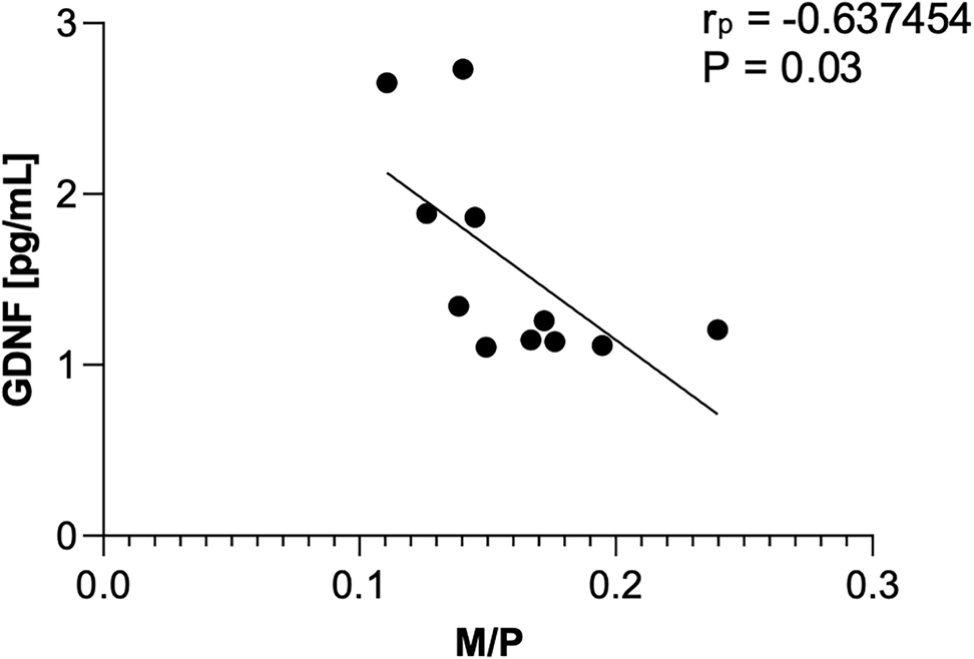
Correlation analysis cerebrospinal fluid (CSF) level of GDNF vs M/P in Progressive Supranuclear Palsy with Richardson’s syndrome (PSP-RS); determined using Pearson’s correlation coefficient (rp).

**Figure 5:**
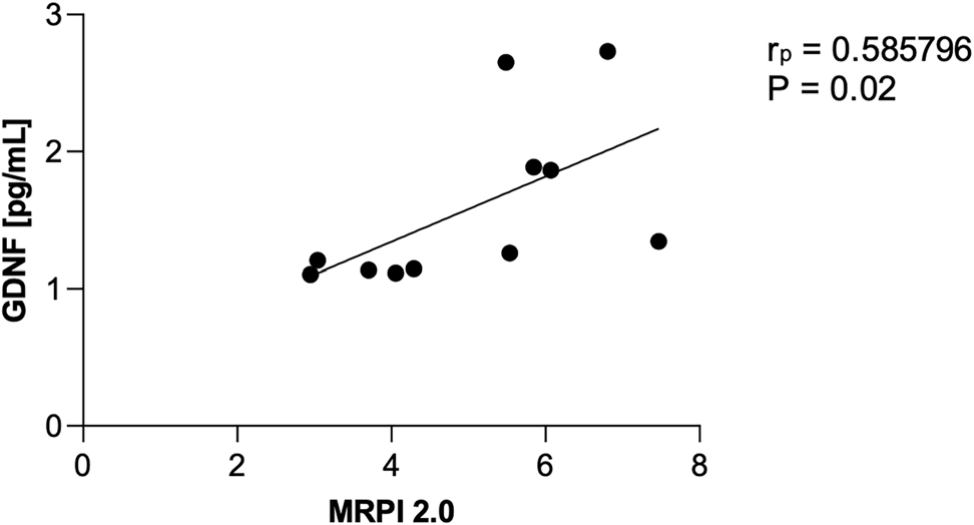
Correlation analysis cerebrospinal fluid (CSF) level of GDNF vs MRPI 2.0 in Progressive Supranuclear Palsy with Richardson’s syndrome (PSP-RS); determined using Pearson’s correlation coefficient (rp).

**Figure 6:**
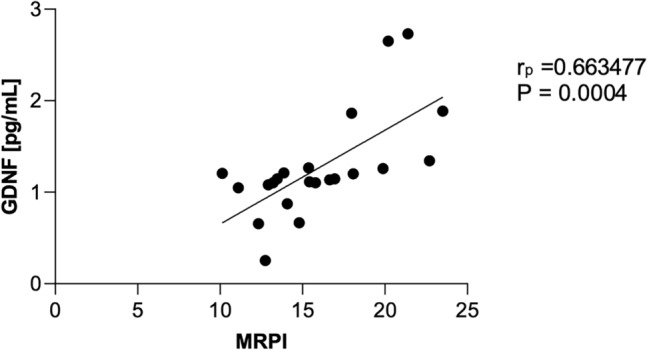
Correlation analysis cerebrospinal fluid (CSF) level of GDNF vs MRPI in Progressive Supranuclear Palsy (PSP); determined using Pearson’s correlation coefficient (rp).

**Figure 7:**
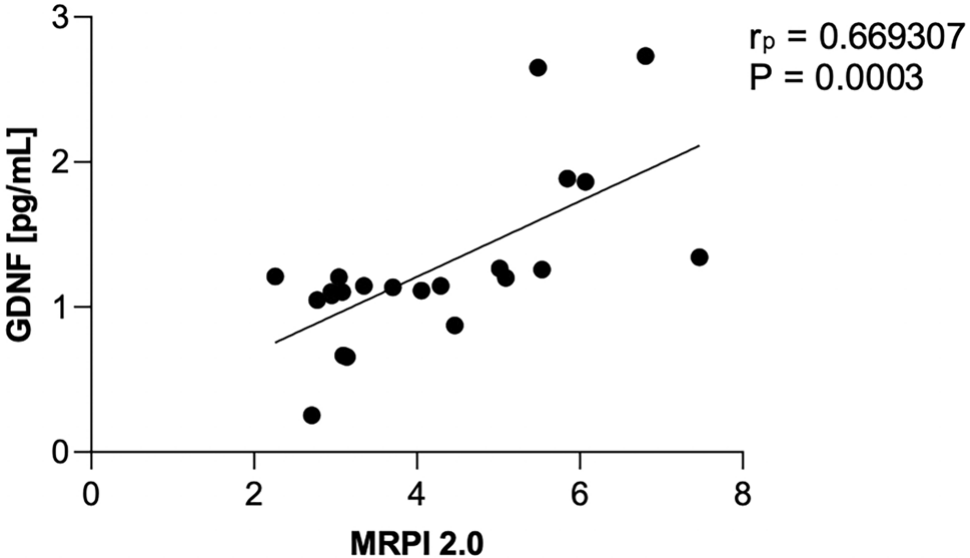
Correlation analysis cerebrospinal fluid (CSF) level of GDNF vs MRPI 2.0 in Progressive Supranuclear Palsy (PSP); determined using Pearson’s correlation coefficient (rp).

**Figure 8:**
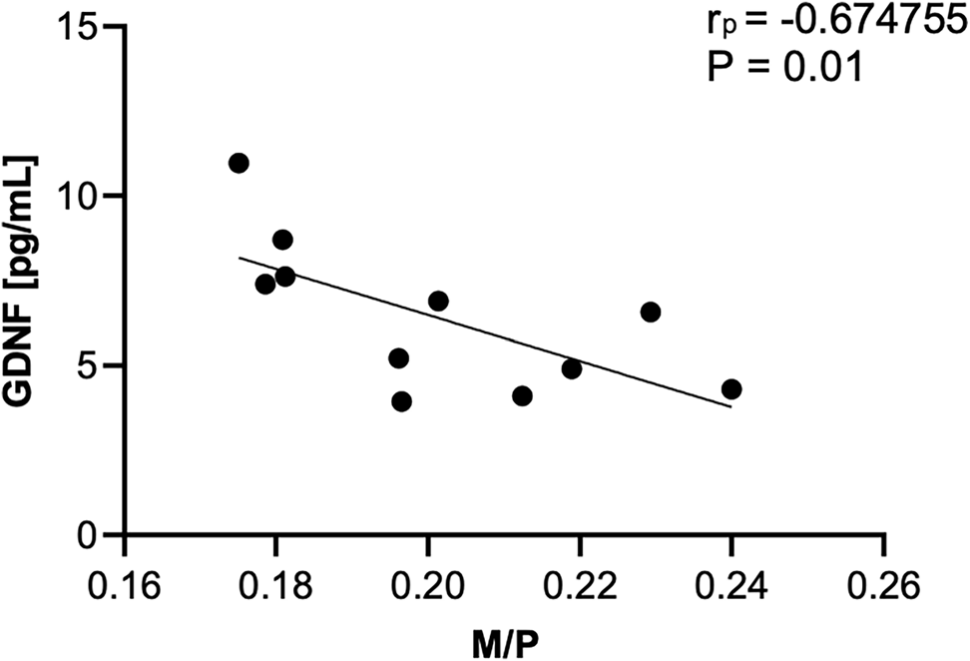
Correlation analysis serum level of GDNF vs M/P in Progressive Supranuclear Palsy with predominant parkinsonism (PSP-P); determined using Pearson’s correlation coefficient (rp).

As shown in Figs. 1 and 2, the analysis of the CSF and in the serum, revealed significantly increased concentrations of GDNF in PSP-RS when compared with PSP-P and control. The CSF GDNF concentration in PSP-RS type (1.68 ± 0.64 pg/ml) was significantly higher than the PSP-P type (0.96 ± 0.2 pg/ml) (Fig. 1). Interestingly in the CSF, the concentrations of GDNF in PSP-P did not significantly differ when compared with healthy volunteers (*P* = 0.504). The serum GDNF concentration of the PSP-RS type (3.45 ± 1.10, *P* < 0.0001) and PSP-P (6.28 ± 1.18,* P* < 0.0001) was significantly higher when compared to healthy volunteers. Additionally, it was observed that the concentration of GDNF in PSP-RS group was statistically significantly lower compared to PSP-P group (*P* < 0.00009) (Fig. 2). Additionally, the concentration of tau in CSF was investigated. The mean tau CSF concentrations did not differ between PSP-RS (2.74 ± 0.82 pg/mL) and PSP-P (2.84 ± 0.85 pg/mL) and were significantly increased when compared to controls (*P* < 0.01; *P* < 0.003). Due to the fact that the control group is younger, authors performed an additional analysis on the correlation between the concentration of GDNF and age, which did not reveal any statistically significant correlation (P > 0.05). Moreover, the evaluations regarding possible correlations between the concentrations of GDNF and the results of FAB and UPDRS-III evaluations did not show any correlation in PSP-RS and PSP-P. The evaluation of tau did not show significant differences between PSP-P and PSP-RS in its levels in the CSF (Fig. 1). Additional evaluation revealed positive correlation between the level of GDNF in the CSF and tau in PSP-P (Fig. 3). The observation was not confirmed in PSP-RS. In both subtypes the levels of tau were significantly increased when compared to healthy volunteers. Authors evaluated possible correlations between the level of GDNF and neuroimaging parameters. GDNF in the CSF was found to be negatively correlated with M/P ratio and positively correlated with MRPI and MRPI 2.0 in PSP-RS (*P* = 0.02–0.04) (Figs. 4, 5). Negative correlation with the area of mesencephalon, M/P ratio and positive correlation with MRPI and MRPI 2.0 was found in the CSF of PSP group without indicating subtypes (*P* =  < 0.001) (Figs. 6, 7). Serum level of GDNF revealed positive correlation with V3 and MRPI 2.0 and negative correlation with M/P ratio and MCP in the PSP-P group (*P* = 0.01–0.03) (Fig. 8).

## Discussion

The role of GDNF in neurodegenerative diseases is not recognized. Its possible impact on the course of two most common phenotypes of PSP seems striking, as in PSP-P the evolution of the disease is not as rapid as in PSP-RS. According to our best knowledge, contemporarily no studies on the role of GDNF in PSP have been published. In PD GDNF is interpreted as a factor which is beneficial in the context of dopaminergic neurons protection^13,14^. In the course of PD, the concentrations of GNDF in the remaining dopaminergic neurons were found to be decreased^5^. In a study based on the examination of 105 patients with PD, authors evaluated the executive functions in PD patients and assessed the GDNF and homovanillic acid concentrations in serum^13^. It was found that the decrease of GDNF is associated with the deterioration of executive functions in PD patients^13^. A different work showed that the decreased concentration of GDNF in the serum is linked with sleep disturbances in PD^15^. In experimental models the levels of GDNF were impacted by the use of rasagiline and deprenyl^14^.

In AD GDNF was found to have a protective role on neurons and glia, additionally in mice models the upregulation of this factor had a protective role in cholinergic transmission, crucial in the pathogenesis of dementia^16^. The levels of GDNF in AD were found to be increased in the CSF, while decreased in the serum^17^. The differences in the levels of GDNF were linked with deviated blood–brain barrier^17^. GDNF was found to impact the transmission within the cortex and striatum upon aging^18^. The levels of GDNF in the serum were considered as a potential factor indicating the stage of AD^19^. The analysis of AD brains revealed the lack of response to this factor in glutaminergic cortical neurons^20^. The possibly protective feature of GDNF was interpreted as a therapeutical possibility in neurodegenerative diseases^21–24^.

The levels of GDNF in PSP, revealed in the study, may suggest a possibly protective role of this factor in a more deteriorating form of the disease, as GDNF release may be activated as a contrary mechanism to accelerated neurodegeneration in PSP-RS. The work does not indicate whether a resembling level of GDNF in the CSF can be observed in PSP-P with a longer disease duration, however the clinical manifestation of advanced stage PSP-P may be an argument in favor of this hypothesis. The significantly increased levels of GDNF in the serum of patients with PSP-P and less increased in PSP-RS may suggest that in PSP, the levels of GDNF in the serum may initially rise significantly as a protective mechanism, which may partly come up with results of studies on GDNF in PD, where the factor inhibited the deterioration of certain clinical features. The verification of tau levels was performed as a factor increasing the veracity of the diagnosis in the light of lack of neuropathological verification.

The manuscript focuses only on PSP-P and PSP-RS, however, based on the results obtained, it could be assumed that in CSF, GDNF level increases in proportion to the severity of atrophy regardless of clinical phenotype. Therefore, in CSF, GDNF level should be higher and more rapidly increasing in phenotypes with more severe brain atrophy and faster clinical deterioration. This marker could be potentially used to evaluate intensity of cerebellar pathology and likely, to monitor effectiveness of future therapies aimed at reducing the pace of disease progression. On the other hand, evaluation of GDNF level in the serum could be used as predictor of upcoming deterioration (indicating the existence of ongoing pathology) and as a marker of effectiveness of neuroprotective mechanisms activated in the course of ongoing neurodegeneration. If the hypothesis presented in the study would be confirmed, and increased GDNF serum level reflects effectiveness of neuroprotection against neurodegeneration, this marker could be used to evaluate future therapies efficacy, as serum GDNF level could be expected to remain increased within successful treatment. Although promising, above hypotheses require thorough further studies to be confirmed.

The differences between the levels of GDNF in serum and CSF of PSP-P and PSP-RS may seem incomprehensible, when taking into account the relatively low specificity of this factor and the unexplored pathomechanism of the diseases. The analysis of the level of GDNF in the CSF in PSP-RS reveals that within the increase of this factor, the MRI assessment of the mesencephalon based on M/P ratio, MRPI and MRPI 2.0 shows more pronounced atrophic changes. This tendency is strongly confirmed (*P* < 0.0005) in the evaluation of PSP group without excluding PSP-P patients, which may suggest that the possible evolution of one PSP subtype to another may be accompanied by changes in the levels of GDNF. Based on the fact that PSP-P and PSP-RS patients were examined at a comparable disease duration, it may be hypothesized that the growth of GDNF in patients with more pronounced atrophic changes within the mesencephalon in PSP-RS is present earlier. In PSP-P, in which the level of GDNF in the serum, is more elevated than in PSP-RS, however in both groups it is above the control level, the level of GDNF is positively correlated with the width of the third ventricle and MRPI 2.0 and negatively correlated with the parameters of MCP and M/P. This, taking into account that generally PSP-RS is associated with more pronounced atrophic changes than PSP-P may suggest than the rise of the GDNF in the serum possibly shows up earlier in PSP-RS than in PSP-P^2^. This may highlight the possibility that in the course of the pathogenesis of PSP, initially the GDNF rise is observed peripherally and consequently within the evolution of other subtypes to PSP-RS, the level of GDNF in the CSF becomes more impacted. The possible mechanism may indicate that patients with PSP-RS as an initial diagnosis, are affected by accelerated transition from “peripheral” to “central” GDNF activation. Moreover the fact that the tau and levels of GDNF in the CSF o PSP-P patients are positively correlated may come up as an argument in favor of subtypes of PSP evolving to PSP-RS.

Based on the assessment of the levels of GDNF in the serum and CSF in the PSP-RS and PSP-P, as well as correlations between the levels of this neurotrophic factor and neuroimaging evaluations of atrophies, the tendency concerning evolution of PSP-P to PSP-RS can be assumed. In the context of advanced PSP-P and PSP-RS, it can be hypothesized that the values of certain parameters among which GDNF could be mentioned may be similar in both subtypes at such stages. Nevertheless despite the clinical resemblance of early PSP-P with PD, searching for possible resemblance of GDNF profile in PSP-P and PD is affected by significant obstacles among which could be mentioned the different pathology and pathomechanism. In the context of advanced PSP-P and PSP-RS these discrepancies cannot be indicated.

The outcome of the study revealed that the level of GDNF is not correlated with FAB results. Regarding results of previous study performed by the same group, which indicated differentiating features of this neuropsychological test in the potential differential examination of PSP-P and PSP-RS, the results of this study show that the concentrations of GDNF are not influential in the context of affecting frontal functions. This may be caused by the fact that other unrecognized factor, possibly depending on the subtype of the disease, may additionally influence the deterioration of frontal functions in the course of PSP. Additional evaluations regarding UPDRS-III revealed that the level of GDNF at this stage should not be linked with the severity of parkinsonian syndromes.

The pattern of pathogenic tau could lead to different phenotypes of PSP, however various phenotypes of the disease lead to the clinical manifestation of PSP-RS. In this context the possible course of atrophies may one hand suggest the path of evolution of the disease, on the other hand may indicate that the growing intensity of atrophic changes throughtout the brain may be linked with gradual evolution to PSP-RS. It was highlighted that PSP is evolving in the anterior to posterior direction. The disease commences in the insula and then passes to the frontal lobe, eventually leading to the temporal, parietal, and occipital lobe^25^. In the context of correlations which were observed between the atrophic changes e.g. in the mesencephalon in the MRI and the growth of GDNF, especially in the evaluations of PSP-RS and PSP (without indication of subtypes), it may be assumed that the growth of GDNF particularly in the CSF is an indicator of tendency towards PSP-RS subtype. In the context of most common phenotypes of PSP—PSP-P and PSP-RS, the pathomechanism may be partly explained by different pace of evolution of clinical symptoms and atrophic changes. In this mechanism GDNF may be interpreted as a factor attempting to inefficiently oppose the primary neurodegenerative heading of PSP. This theory may be affected by limitations when discussed in the context of very rare phenotypes mentioned in the criteria of diagnosis and very briefly in contemporary literature e.g.—PSP-with predominant frontal presentation (PSP-F), PSP with predominant oculomotor dysfunction (PSP-OM), PSP with predominant cerebellar dysfunction (PSP-C) etc. Further evaluation of these groups of patients may be explored after evaluation of broader groups of patients.

### Limitations

The study is affected by certain limitations. No neuropathological verification was done, as all of the patients included in the study remain alive. As a partial supplementary verification, authors verified the concentrations of tau in the CSF, which was significantly higher in PSP-RS and PSP-P when compared to controls and similar PSP-RS and PSP-P. The groups are small as Authors intended to perform the differential diagnosis of two most common phenotypes of PSP. The GDNF measurements presented in this study were performed only once, no follow-up evaluation was done. Additionally, authors analyzed only one factor possibly impacting the subtypes of the disease in two types of samples. The clinical evaluation was performed using UPDRS-III which is not dedicated to evaluation of PSP, as the PSP-Rating Scale was not validated in Polish. The control group is younger than the examined groups, however the evaluation of the control group did not reveal any correlation between the concentration of GDNF and age.

## Conclusion

GDNF is a possibly promising factor in the context of future therapies, which seems especially intriguing in the context of entities lacking effective treatment. The data on the role of GDNF in PSP is limited in contemporary literature, however the outcomes of studies on AD and PD patients may suggest that GDNF may be a feature impacting the course and stage of PSP. The factor may be feasible in the treatment of this disease as it may evolve as a feature inhibiting its course. Due to the limited information on the role of GDNF in PSP, more data in the field is required.

## Data Availability

The data supporting the findings of this study are available on request from the corresponding author. The data are not publicly available due to privacy or ethical restrictions.
